# Non-contiguous finished genome sequence and description of *Sulfurimonas hongkongensis* sp. nov., a strictly anaerobic denitrifying, hydrogen- and sulfur-oxidizing chemolithoautotroph isolated from marine sediment

**DOI:** 10.4056/sigs.4948668

**Published:** 2014-02-15

**Authors:** Lin Cai, Ming-Fei Shao, Tong Zhang

**Affiliations:** 1 Environmental Biotechnology Laboratory, Department of Civil Engineering, The University of Hong Kong, Hong Kong SAR, China; 2 Department of Civil and Environmental Engineering, Harbin Institute of Technology Shenzhen Graduate School, Shenzhen, China

**Keywords:** *Sulfurimonas hongkongensis*, chemolithoautotroph, sulfur oxidation, denitrification, anaerobe, marine sediment, genome

## Abstract

Here, we report a type strain AST-10 representing a novel species *Sulfurimonas hongkongensis* within *Epsilonproteobacteria*, which is involved in marine sedimentary sulfur oxidation and denitrification. Strain AST-10^T^ (= DSM 22096^T^ = JCM 18418^T^) was isolated from the coastal sediment at the Kai Tak Approach Channel connected to Victoria Harbour in Hong Kong. It grew chemolithoautotrophically using thiosulfate, sulfide or hydrogen as the sole electron donor and nitrate as the electron acceptor under anoxic conditions. It was rod-shaped and grew at 15-35°C (optimum at 30°C), pH 6.5-8.5 (optimum at 7.0-7.5), and 10-60 g L^-1^ NaCl (optimum at 30 g L^-1^). Genome sequencing and annotation of strain AST-10^T^ showed a 2,302,023 bp genome size, with 34.9% GC content, 2,290 protein-coding genes, and 42 RNA genes, including 3 rRNA genes.

## Introduction

The genus *Sulfurimonas* was formally proposed in 2003, and included only one species, *Sulfurimonas autotrophica* OK10^T^, at that time [1]. Since then, several novel species have been identified, such as *Sulfurimonas paralvinellae* GO25^T^ [2], *Sulfurimonas denitrificans* DSM 1251^T^ (reclassified, previously known as *Thiomicrospira denitrificans*) [2], and *Sulfurimonas gotlandica* GD1^T^ [3]. Here, we report another novel species, *Sulfurimonas hongkongensis* AST-10^T^, isolated from coastal sediment, and describe its features, together with the genome sequencing and annotation.

Currently, all known *Sulfurimonas* members were isolated from marine sediments except for strain GD1 from deep seawater [4]. The most widely shared feature of *Sulfurimonas* members is chemolithoautotrophy; strains can grow by oxidizing hydrogen gas, elemental sulfur, hydrogen sulfide, or thiosulfate [1-7]. In our previous studies, anoxic sulfur-oxidizing bacteria were demonstrated to dominate the nitrate induced marine sediment remediation process [8-10]. Phylogenetic analysis based on 16S rRNA genes showed that *Epsilonproteobacteria* closely related to *S. denitrificans* constituted the major bacterial population during such remediation of the sediment at Kai Tak Approach Channel, Hong Kong, China. Strain AST-10^T^ was isolated from the sediment and named *Sulfurimonas hongkongensis* sp. nov., based on its unique physiological and phylogenetic characteristics.

## Classification and features

Sediment was collected 10-50 cm below the seawater/sediment interface at the Kai Tak Approach Channel connected to Victoria Harbor in Hong Kong, China. Sewage and industrial effluent had been discharged there for decades until the installation of a new sewage collection system in the late 1990s. The long lasting sulfate-reducing conditions resulted in a high sulfide concentration in the sediment, where an AVS (Acid-Volatile Sulfide) of 198 μmol g^-1^ had been measured [8]. The pore water after centrifugation at 4,000 rpm for 15 min had a pH of 7.89 and a salinity of 2.9%.

Enrichments were prepared by adding 20 g of wet sediment (32.0% dry matter) to serum bottles containing 70 mL of sterilized seawater, purged with N_2_ and incubated for at least 24 h at room temperature. Potassium nitrate (1 g L^-1^) and sodium phosphate, monobasic (0.1 mmol L^-1^), were then added from sterilized stock solutions. The bottles were incubated at 28°C in a water bath for 72 h. The enrichments were plated onto agar plates of DSM113-S medium, a salinity modified version of DM113 medium that is recommended by DSMZ for nitrate-reducing and sulfide-oxidizing bacteria. One liter of DSM113-S contained: KH_2_PO_4_ (2.0 g), KNO_3_ (4.0 g), NH_4_Cl (1.0 g), MgSO_4_·7H_2_O (0.8 g), Na_2_S_2_O_3_·5H_2_O (5.0 g), NaHCO_3_ (1.0 g), FeSO_4_·7H_2_O (2.0 mg), NaCl (25.0 g) and 2 ml of trace element solution SL-4. Solid media contained 1.5% bacterial agar from Difco. All media were sterilized by autoclaving and cooled under N_2_ atmosphere. Colonies formed on plates were picked and further purified by re-streaking single colonies on agar plates for more than 20 rounds (4-10 d round^-1^). A colony isolated and purified from the above process was defined as strain AST-10^T^.

**Figure 1:**
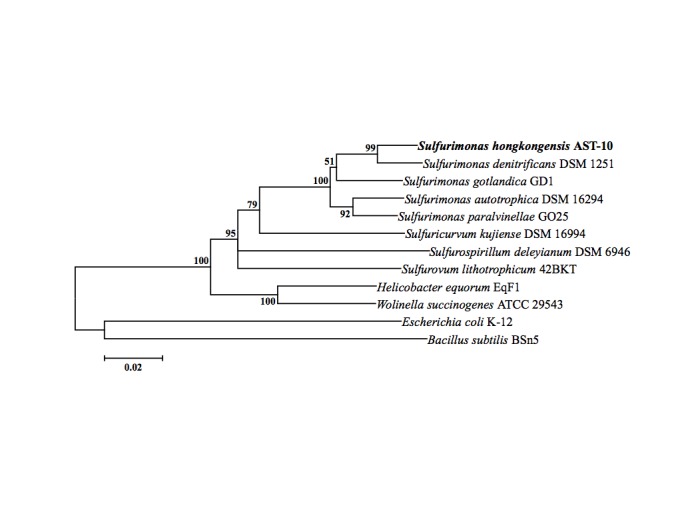
Phylogenetic tree highlighting the position of *Sulfurimonas hongkongensis* relative to the other species within the *Helicobacteriaceae*. The neighbor-joining tree was constructed using MEGA 5.05 and tested with 1,000 bootstrap replicates. Bootstrap values over 50% are shown and the scale bar 0.02 represents 2% nucleotide substitution. All reference sequences can be exactly searched and retrieved from NCBI GenBank based on the full name of each strain.

The 16S phylogenetic tree shown in Figure 1 indicated that strain AST-1^T^ is a member of the genus *Sulfurimonas*, (Table 1). An online BLAST query in NCBI using the 16S rRNA gene sequence from strain AST-1^T^ showed a relatively low identity to all currently identified *Sulfurimonas* species, including *S. denitrificans* DSM 1251^T^ (97% identity), *S. gotlandica* GD1^T^ (95% identity), *S. autotrophica* OK10^T^ (95% identity), and *S. paralvinellae* GO25^T^ (94% identity). Using the commonly accepted criterion of a 97% 16S rDNA sequence similarity cut-off for defining species [19,20], strain AST-10^T^ could accordingly be identified as a novel species within the genus *Sulfurimonas*.

**Table 1:** Classification and general features of *Sulfurimonas hongkongensis* AST-10 based on the MIGS recommendations [11]

**MIGS ID**	**Property**	**Term**	**Evidence code**^a^
	Current classification	Domain *Bacteria*	TAS [12]
		Phylum *Proteobacteria*	TAS [13]
		Class *Epsilonproteobacteria*	TAS [14,15]
		Order *Campylobacterales*	TAS [14,16]
		Family *Helicobacteraceae*	TAS [14,17]
		Genus *Sulfurimonas*	TAS [1-3]
		Species *Sulfurimonas hongkongensis*	IDA
		Type strain AST-10	IDA
	Gram stain	Gram-negative	TAS [1]
	Cell shape	Rod-shaped, 0.2-0.4 µm x 0.5-1.2 µm	IDA
	Motility	Not reported	
	Sporulation	No	NAS
	Temperature range	15-35^o^C	IDA
	Optimum temperature	30°C	IDA
	Carbon source	HCO_3_^-^, CO_2_	IDA
	Energy source	H_2_, HS^-^ or S_2_O_3_^2-^	IDA
	Terminal electron receptor	NO_3_^-^	IDA
MIGS-6	Habitat	Coastal sediment	IDA
MIGS-6.3	Salinity	10-60 g L^-1^ NaCl, optimum at 30 g L^-1^	IDA
MIGS-22	Oxygen	Strict anaerobe	IDA
MIGS-15	Biotic relationship	Free living	IDA
MIGS-14	Pathogenicity	Not reported as a pathogen	NAS
MIGS-4	Geographic location	Kai Tak Approach Channel, Hong Kong	IDA
MIGS-5	Sample collection time	July, 2006	IDA
MIGS-4.1	Latitude	22.33°N	TAS
MIGS-4.2	Longitude	114.19°E	TAS
MIGS-4.3	Depth	10-50 cm depth of coastal sediment	IDA
MIGS-4.4	Altitude	below sea surface	IDA

a) Evidence codes - IDA: Inferred from Direct Assay; TAS: Traceable Author Statement (i.e., a direct report exists in the literature); NAS: Non-traceable Author Statement (i.e., not directly observed for the living, isolated sample, but based on a generally accepted property for the species, or anecdotal evidence). These evidence codes are from the Gene Ontology project [18].

**Figure 2:**
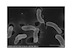
Scanning electron micrograph of *Sulfurimonas hongkongensis* AST-10^T^. The scale bar represents 1.0 µm.

Cell morphology was examined by Scanning Electron Microscopy (SEM). As shown in Figure 2, the cells of AST-10^T^ were rod-shaped, 0.2-0.4 μm in diameter, and 0.5-1.2 μm in length. On solid medium, AST-10^T^ grew and formed small, white, transparent, round shaped colonies with smooth boundaries.

Physiology

Effects of temperature, pH, and salinity on the growth of strain AST-10^T^ were investigated, showing that it grew at 15-35°C (optimum at 30°C), pH 6.5-8.5 (optimum at 7.0-7.5), and 10-60 g L^-1^ NaCl (optimum at 30 g L^-1^). The generation time of strain AST-10^T^ under optimal conditions was tested as 6.1 h. It was significantly shorter than other species, such as *S. paralvinellae* GO25^T^ and *S. denitrificans* DSM 1251^T^. The cell yield of strain AST-10^T^ was 5.2 g dry weight per mole of S_2_O_3_^2-^. This value is similar to that of its *Epsilonproteobacterial* relative *S. denitrificans* DSM 1251^T^ (5.72 g), but only about one-half of the *Betaproteobacterial Thiobacillus denitrificans* (11.6 g). Such difference in growth efficiency might be attributed to the different pathways used for carbon fixation and metabolism.

To determine whether electron acceptors other than NO_3_^-^ would sustain the growth of strain AST-10^T^, SO_4_^2-^, NO_2_^-^, Fe^3+^, and O_2_ were separately tested with S_2_O_3_^2-^ as the sole electron donor. No growth was observed using any of these electron acceptors. S_2_O_3_^2-^, HS^-^, and H_2_ can support the growth of strain AST-10^T^ as electron donors, however, acetate, lactate, malate, formate, pyruvate, glucose, glycerol, and yeast extract cannot. Hence, strain AST-10^T^ was a chemolithoautotroph, using NO_3_^-^ as an electron acceptor and S_2_O_3_^2-^, HS^-^, or H_2_ as an electron donor. The time course of S_2_O_3_^2-^ oxidation and NO_3_^-^ reduction during strain AST-10^T^ growth was monitored. N_2_ was the dominant denitrification product, no accumulation of N_2_O and NO_2_^-^ was detected, when it was cultivated using DSM113-S at 30°C and pH 7.5. Significant production of insoluble S^0^ occurred when it was cultured with an excess amount of S_2_O_3_^2-^ (molar ratio of S_2_O_3_^2-^/NO_3_^-^ > 2). SO_4_^2-^ became the dominant oxidation product under excess NO_3_^-^ conditions (molar ratio of S_2_O_3_^2-^/ NO_3_^-^ < 0.25). This was quite similar to the well-characterized strain *Thiomicrospira* CVO [21]. But for *S. denitrificans* DSM 1251^T^, no accumulation of insoluble S^0^ was observed even under a high molar ratio of S_2_O_3_^2-^/NO_3_^-^ [5].

### Chemotaxonomy

Cellular fatty acid composition was analyzed using the cells grown in DSM113-S medium at 30°C in the late-exponential phase. The major cellular fatty acids of strain AST-10^T^ were C_14:0_ (4.8%), C_16:0_ (32.8%), 2-OH C_16:0_ (9.5%), C_16:1_ (14.6%), C_18:0_ (16.9%), and C_18:1_ (19.2%). This composition was generally similar to those of *S. paralvinellae* GO25^T^ and *S. autotrophica* OK10^T^. However, 2-OH C_16:0_ was a unique fatty acid, differentiating AST-10^T^ from other species within the genus of *Sulfurimonas*.

## Genome sequencing and annotation

### Genome project history

The strain was selected for genome sequencing on the basis of its 16S rRNA gene-based phylogenetic position within the genus *Sulfurimonas* (Table 1). It is the first sequenced genome of *Sulfurimonas hongkongensis* sp. nov. A summary of the genome sequencing project information is shown in Table 2. The genome consists of 28 contigs, which has been deposited at DDBJ/EMBL/GenBank under accession number AUPZ00000000. The version described in the present study is the first version.

**Table 2:** Genome sequencing project information

**MIGS ID**	**Property**	**Term**
MIGS-31	Finishing quality	High-quality draft
MIGS-28	Libraries used	Paired-end 500 bp shotgun library
MIGS-29	Sequencing platforms	Illumina HiSeq 2000
MIGS-31.2	Fold coverage	3,011 ×
MIGS-30	Assemblers	CLC Genomics Workbench 6.0.2
MIGS-32	Gene calling method	GeneMarkS+
	Genbank ID	AUPZ00000000
	Genbank date of release	August 13, 2013
	Project relevance	Ecology and Evolution

### Growth conditions and DNA isolation

As described above, the strain was grown in DSM113-S medium under anoxic condition with optimal growth at 30^o^C, pH7.0-7.5, and NaCl 30 g L^-1^. The genomic DNA used for shotgun sequencing was prepared by DSMZ.

### Genome sequencing and assembly

The genome shotgun sequencing project was finished by BGI (Beijing Genomics Institute). Briefly, DNA was first mechanically fragmented with an enrichment size of ~500 bp. Then the DNA fragmentation was gel purified and quality checked. The recycled DNA was used for shotgun library construction, which was finally sequenced on an Illumina HiSeq 2000 platform using the paired-end 150 bp sequencing strategy.

A total of 6,932,096,700 bp of raw sequence was obtained, which was assembled with CLC Genomics Workbench 6.0.2 using a word size of 40 bp. The draft genome was finally assembled into 28 contigs with a 2,302,023 bp genome size and more than 3,000 fold genome coverage (Table 3).

**Table 3:** Nucleotide content and gene count levels of the genome

**Attribute**	**Value**	% of total
Genome size (bp)	2,302,023	100%
DNA coding region (bp)	2,127,855	92.4%
DNA G+C content (bp)	803,203	34.9%
Number of contigs	28	
Contig N50 (bp)	235,215	
Total genes^b^	2332	100%
RNA genes	42	1.8%
rRNA genes	3	0.1%
tRNA genes	39	1.7%
Protein-coding genes	2290	98.2%
Pseudo genes	0	0.0%
Frameshifted genes	0	
Protein-coding genes with function prediction	1146	50.0%
Protein-coding genes assigned to COGs	1700	74.2%
Protein-coding genes assigned Pfam domains	1516	66.2%
Protein-coding genes with signal peptides	155	6.8%
Protein-coding genes with transmembrane helices	565	24.7%

a) The total is based on either the size of the genome in base pairs or the total number of protein coding genes in the annotated genome.

b) Also includes 54 pseudogenes and 5 other genes.

### Genome annotation

The draft genome was annotated by NCBI Prokaryotic Genome Annotation Pipeline (PGAP). Protein-coding genes with function prediction were calculated based on the PGAP result. The COGs (Clusters of Orthologous Groups) functional annotation was conducted by PRSBLAST search against COGs database with an E-value cutoff 1e-10 [22,23]. Pfam domains were annotated using HMMER 3.0 program on Pfam database with an E-value cutoff 1e-10 [24,25]. SignalP 4.1 Server was employed to analyze proteins with signal peptide [26]. TMHMM Server 2.0 was used to predict transmembrane helices in proteins [27].

## Genome properties

**Figure 3:**
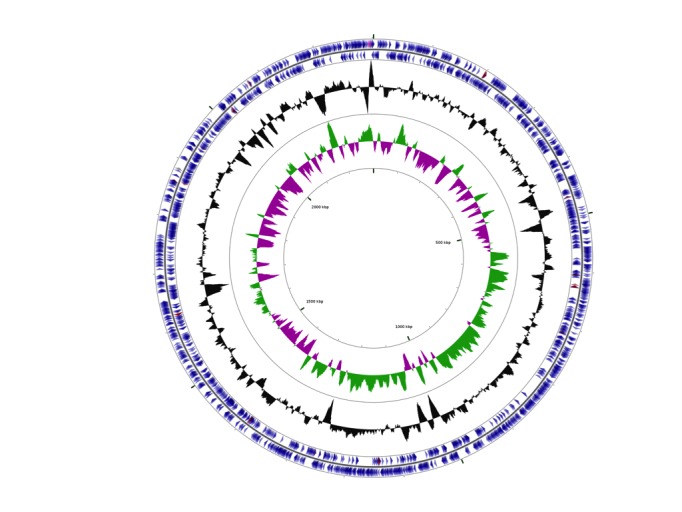
Graphical circular map of the *Sulfurimonas hongkongensis* AST-10 genome. Seen from the outside to the inside: genes on forward strand, genes on reverse strand, GC content, GC skew. The graphical map was plotted on the CGview Server.

The draft genome of *Sulfurimonas hongkongensis* AST-10^T^ was assembled into 28 contigs with a total size of 2,302,023 bp and a GC content of 34.9%. 2,332 genes were annotated, 2,290 of which were protein-coding genes. The remaining 42 genes were RNA genes including 3 rRNA genes. A total of 1,146 of the protein-coding genes were assigned putative functions. The remaining 1,144 protein-coding genes were annotated as hypothetical proteins. The AST-10^T^ genome properties and statistics are summarized in Tables 2-4 and Figure 3.

**Table 4:** Number of genes associated with the 25 general COG functional categories

**Code**	**Value**	**%age**^a^	**Description**
J	130	5.7	Translation
A	0	0.0	RNA processing and modification
K	64	2.8	Transcription
L	89	3.9	Replication, recombination and repair
B	0	0.0	Chromatin structure and dynamics
D	16	0.7	Cell cycle control, mitosis and meiosis
Y	0	0.0	Nuclear structure
V	27	1.2	Defense mechanisms
T	163	7.1	Signal transduction mechanisms
M	138	6.0	Cell wall/membrane biogenesis
N	68	3.0	Cell motility
Z	0	0.0	Cytoskeleton
W	0	0.0	Extracellular structures
U	58	2.5	Intracellular trafficking and secretion
O	69	3.0	Posttranslational modification, protein turnover, chaperones
C	128	5.6	Energy production and conversion
G	52	2.3	Carbohydrate transport and metabolism
E	134	5.9	Amino acid transport and metabolism
F	55	2.4	Nucleotide transport and metabolism
H	97	4.2	Coenzyme transport and metabolism
I	42	1.8	Lipid transport and metabolism
P	101	4.4	Inorganic ion transport and metabolism
Q	17	0.7	Secondary metabolites biosynthesis, transport and catabolism
R	158	6.9	General function prediction only
S	94	4.1	Function unknown
-	590	25.8	Not in COGs

a) The total is based on the total number of protein coding genes in the annotated genome.

## Conclusion

### Description of *Sulfurimonas hongkongensis* sp. nov.

*Sulfurimonas hongkongensis* (hong.kong.en'sis. N.L. fem. adj. *hongkongensis* pertaining to Hong Kong, the city where the type strain was isolated).

Strain AST-10^T^ is rod-shaped with size of 0.2-0.4 µm x 0.5-1.2 µm. It is an obligate anaerobe and occurs singly. The temperature range for growth is 15-35^o^C, optimum at 30^o^C. The pH range for growth is 6.5-8.5, optimum at 7.0-7.5. The salinity range for growth is 10-60 g L^-1^, and optimum at 30 g L^-1^. Strictly chemolithoautotrophic growth occurs with H_2_, HS^-^ or S_2_O_3_^2-^ as an electron donor and with nitrate as an electron acceptor. Nitrate is reduced to N_2_, and reduced sulfur compounds are oxidized into S^0^ or SO_4_^2-^ (depending on molar ratio of S_2_O_3_^2-^/NO_3_^-^). The major cellular fatty acids are C_14:0_, C_16:0_, 2-OH C_16:0_, C_16:1_, C_18:0_, and C_18:1_, with C_16:0 2-OH_ as a unique fatty acid different from other species in the genus *Sulfurimonas*.

The type strain AST-10^T^ = DSM 2096^T^ = JCM 18418^T^, was isolated from coastal sediment at the Kai Tak Approach Channel connected to Victoria Harbour in Hong Kong, China. The GC content of the genome is 34.9%. The genome sequence has been deposited at DDBJ/EMBL/GenBank under accession number AUPZ00000000.
